# Music in disorders of consciousness

**DOI:** 10.3389/fnins.2014.00190

**Published:** 2014-07-03

**Authors:** Jens D. Rollnik, Eckart Altenmüller

**Affiliations:** ^1^BDH-Clinic Hessisch Oldendorf, Teaching Hospital of Hannover Medical School (MHH), Institute for Neurorehabilitational Research (InFo)Hessisch Oldendorf, Germany; ^2^Institute of Music Physiology and Musician's Medicine (MMM), University of Music, Drama and Media HannoverHannover, Germany

**Keywords:** music therapy, music, coma, unresponsive wakefulness syndrome, minimally conscious state

## Abstract

This review presents an overview of the use of music therapy in neurological early rehabilitation of patients with coma and other disorders of consciousness (DOC) such as unresponsive wakefulness syndrome (UWS) or minimally conscious state (MCS). There is evidence that patients suffering from UWS show emotional processing of auditory information, such as listening to speech. Thus, it seems reasonable to believe that music listening—as part of an enriched environment setting—may be of therapeutic value in these patients. There is, however, a considerable lack of evidence. The authors strongly encourage further studies to evaluate the efficacy of music listening in patients with DOC in neurological early rehabilitation. These studies should consider a precise clinical definition and homogeneity of the patient cohort with respect to the quality (coma vs. UWS vs. MCS), duration (rather weeks to months than days) and cause (traumatic vs. non-traumatic) of DOC, a standardized intervention protocol, valid clinical outcome parameters over a longer observation period (weeks to months), monitoring of neurophysiological and vegetative parameters and, if available, neuroimaging to confirm diagnosis and to demonstrate responses and functional changes in the patients' brains.

## Introduction

Rehabilitation of patients with stroke, hypoxic encephalopathy or severe brain injury is challenging. When considering music as therapy in neurological rehabilitation, one should be aware that there are two distinct groups of patients: First, early rehabilitation patients, frequently comatose (or suffering from other disorders of consciousness). They have a low functional status, high morbidity and are dependent on nursing (Rollnik and Janosch, 2010; Rollnik, 2011, 2014), requiring “passive” therapies (rather listening to music than playing). Second, patients at subsequent stages of rehabilitation, aware, with improving functional status, gaining independence from nursing. These patients require more and more “active” therapies. Along with improvement of consciousness and functional status, their ability to cooperate increases and they may participate in more active therapies (rather playing than listening to music).

The present review focuses on the efficacy of music as a therapeutic tool in early rehabilitation patients with disorders of consciousness (DOC). In Germany, neurological and neurosurgical patients are transferred to specialized early neurological rehabilitation centers, immediately after acute hospital treatment (e.g., brain surgery) (Rollnik and Janosch, 2010; Rollnik, 2011). These centers offer intensive care unit treatment because early rehabilitation patients need to be monitored and are frequently dependent on mechanical ventilation (Rollnik and Janosch, 2010; Rollnik, 2011).

Before reviewing the efficacy of music in early rehabilitation, a precise definition of DOC appears to be useful. First of all, coma is a clinical syndrome characterized by reflex behavior and a disorder of consciousness, no eye opening even to strong painful stimuli may be observed (Bodard et al., 2013). In the unresponsive wakefulness syndrome (UWS)—previously known as vegetative state (VS)—, eyes are open and reflex behavior occurs, but patients are completely unresponsive (e.g., absence of command following) (Bodard et al., 2013). Patients in a minimally conscious state (MCS) can show signs of consciousness, such as command following (even if inconsistent), visual pursuit, localization to noxious stimulation, and appropriate responses to emotional stimuli without being able to functionally communicate (Bodard et al., 2013). It has been suggested to distinguish two groups of MCS patients: Those who show higher-order signs of consciousness as MCS+ (e.g., non-functional communication and command following) from MCS− with only low-level signs of consciousness (e.g., visual pursuit, noxious stimulation localization, appropriate emotional response) (Bodard et al., 2013). These DOC have to be separated from the locked-in syndrome (LIS) which can be found in brain-stem injured patients and is characterized by preserved cognition and eye-coded communication (eye movements) with a lack of any further motor output (Bodard et al., 2013). It has also been suggested to define a functional LIS (fLIS) describing patients with severe brain injury who are behaviorally in an UWS or MCS, but on neuroimaging show better consciousness than expected, with command following and even functional communication) (Bodard et al., 2013). Table 1 summarizes the clinical features of coma and other DOC.

**Table 1:** **Clinical diagnostic criteria of disorders of consciousness (DOC) (Bodard et al., 2013)**.

	**Coma**	**Unresponsive wakefulness syndrome (UWS)**	**Minimally conscious state (MCS)**	
			**MCS−**	**MCS+**
Reflex behavior	+	+	−	−
Eye opening	−	+	+	+
Functional communication	−	−	−	−
Low-level signs of consciousness^a^	−	−	+	+
High-level signs of consciousness^b^	−	−	−	+

a Visual pursuit, noxious stimulation localization, appropriate emotional response.

b Command following, non-functional communication.

We know that listening to music influences mood and arousal, which may improve performance on a variety of cognitive tasks (called the “Mozart effect” or “mood and arousal hypothesis”) (Husain et al., 2002). While musical tempo affects arousal, mode (major or minor) may change mood (Husain et al., 2002). There is broad evidence that mood plays a major role in neurological rehabilitation, mood improvement is associated with functional recovery of stroke patients, for instance Bilge et al. (2008). Music listening may be used to facilitate the recovery of cognitive functions and mood after stroke (Särkämö et al., 2008). Listening to self-selected music (at least 1 h daily for 2 months) improved verbal memory, focused attention, depressed, and confused mood (Särkämö et al., 2008). It is reasonable to believe that music listening may be of therapeutic value in neurological rehabilitation of patients without DOC. Improvement of mood and attention seems to be the key component of this “Mozart effect.” However, it is unclear whether music listening has any therapeutic effect in DOC. The present review examines if music—through emotional and other processes (e.g., arousal)—might be able to improve consciousness in these patients. To understand its potential it is helpful to focus on some neurobiological aspects of listening in healthy subjects and DOC.

## Neurobiological aspects of listening in DOC patients

**Figure 1:**
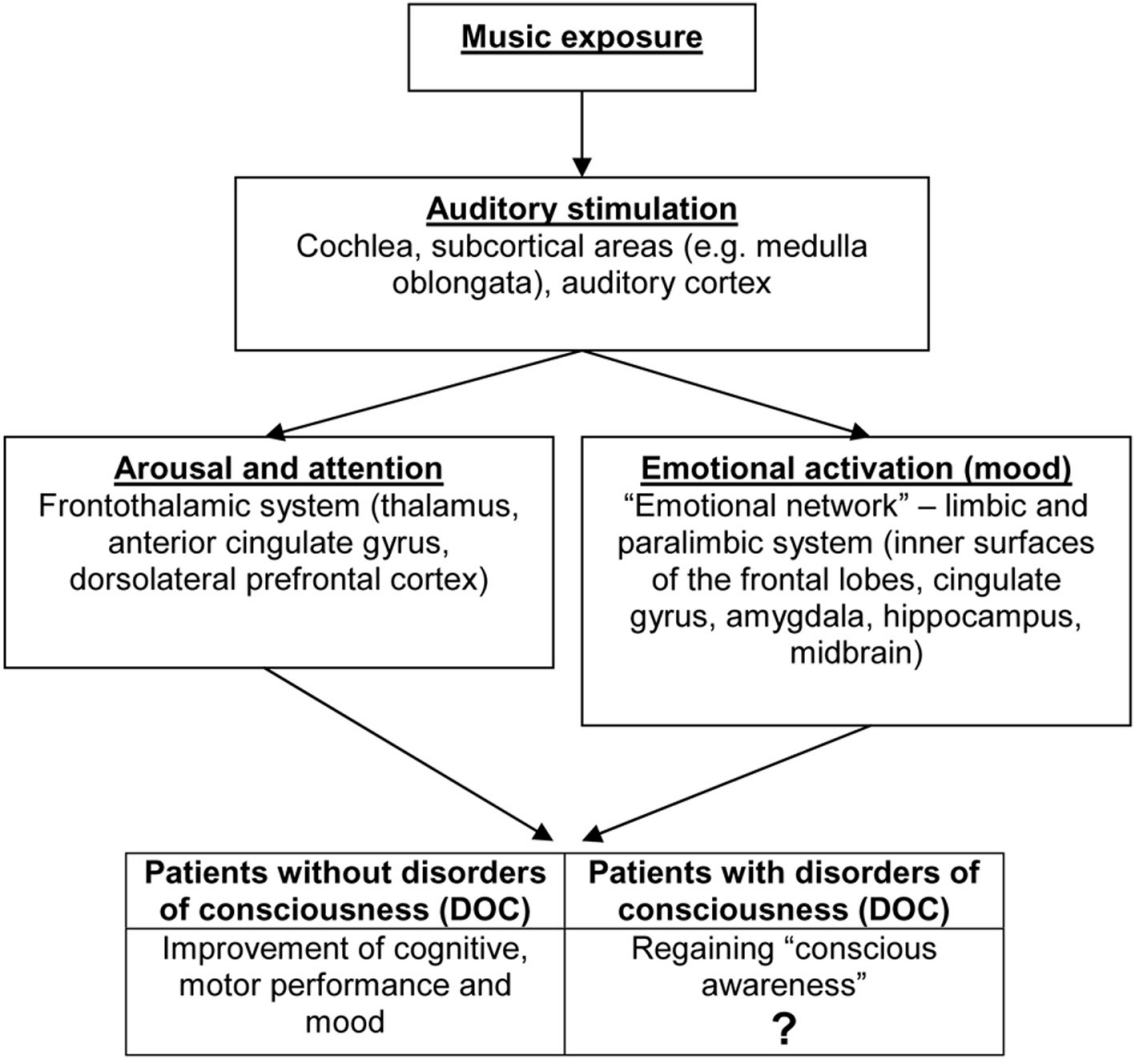
**Music processing**. Potential effects of music listening and brain structures involved (Altenmüller and Schlaug, 2013). Strongly simplified model based on the “mood and arousal hypothesis” (Husain et al., 2002).

Listening to music induces a widespread cortical and subcortical activation of the brain (Altenmüller and Schlaug, 2013). A strongly simplified model of music processing, potential effects of music listening and brain structures involved is presented in Figure 1. The model is based on the “mood and arousal hypothesis” which has been described above (Husain et al., 2002).

It has been shown in neuroimaging studies that music listening activates a vast bilateral network of temporal, frontal, parietal, cerebellar and limbic structures related to attention, semantic processing, memory and the motor system (Särkämö et al., 2008; Altenmüller and Schlaug, 2013). Besides speech, music is the most versatile and complex auditory experience integrating input from the auditory, visual, and somatosensory system (Altenmüller and Schlaug, 2013). In addition, the basis and inner surfaces of the frontal lobes, the cingulate gyrus, amygdala, hippocampus and midbrain are involved in the emotional perception of music (Peretz and Zatorre, 2005; Altenmüller and Schlaug, 2013). For a detailed review on neurobiological aspects of music listening, see Peretz and Zatorre (2005), Altenmüller and Schlaug (2013).

So far, music listening seems to be advantageous for alert healthy subjects (Husain et al., 2002). It may stimulate the emotional network and improve attention and cognitive performance. But how about patients with DOC, do they respond to auditory or any other stimulation at all? Recently, it has been shown that patients with UWS do respond to pain cries of other people (Yu et al., 2013). These patients showed an activation of the so-called pain matrix, involving a sensory subsystem (which underlies pain sensation) and an affective subsystem (which underlies aversive emotional pain effects) (Yu et al., 2013). We know from other neuroimaging studies (functional magnetic resonance imaging—fMRI) that UWS patients may have cortical responses to language stimulation (Coleman et al., 2007). It has even been demonstrated that familiar speakers evoked significantly stronger activation in the limbic system (amygdala) than unfamiliar speakers and neutral phrases (Eickhoff et al., 2008). These findings indicate that listening to familiar sounds may not only induce cognitive but also emotional processing in UWS (Eickhoff et al., 2008). Visual stimuli are emotionally processed in UWS patients too (Sharon et al., 2013). Patients displayed more pronounced limbic and cortical activations elicited by presentation of familiar than non-familiar faces (Sharon et al., 2013). The fact that limbic and cortical areas have been activated supports the hypothesis that these responses might be a sign of “heightened awareness.” The finding of brain responses to emotional stimuli in patients with UWS is of importance because the quality of awareness cannot be evaluated without addressing the question of whether cognitive processes also elicit a subjective emotional experience (Sharon et al., 2013). Emotion and consciousness are considered to be inseparable as each conscious state is endowed with some form of emotion, for a detailed review, see Berkovich-Ohana and Glicksohn (2014). Emotion is regarded as a key component of our experiencing of environment, including our sense of self, serving as an ever-present basic constitute of the quality of human consciousness (Sharon et al., 2013).

## Therapeutic approaches in patients with DOC (multisensory stimulation)

It has been hypothesized that comatose patients might suffer from a condition of “environmental deprivation” (LeWinn and Dimancescu, 1978). This condition could be improved by environmental inputs through all five sensory pathways enhancing the rate and degree of recovery from coma (LeWinn and Dimancescu, 1978). The idea of “enriched environment” inspires therapeutic approaches using sensory stimulation in neurological early rehabilitation (Lippert-Grüner et al., 2007).

A Cochrane systematic review focused on sensory stimulation of brain-injured patients with coma or UWS (Lombardi et al., 2002). The authors identified only three studies which met the well-defined inclusion criteria (coma or UWS patients, brain injury of traumatic or non-traumatic origin, randomized controlled and non-randomized controlled trials with concurrent controls, comparing sensory stimulation with standard rehabilitation): In one randomized controlled study (RCT), only seven comatose patients (admitted to the ICU within 24 h after traumatic brain injury due to road traffic accident) in the intervention group underwent a multisensory stimulation of all five senses (olfactory, visual, auditory, gustatory, tactile) 20 min per day during their stay on the ICU (medium stay 8.1 days) (Johnson et al., 1993). There was no such stimulation in the control group. Outcome measures were Glasgow Coma Scale (GCS), ventilation, brain stem reflexes, spontaneous eye movements, skin conductance and heart rate assessed 20 min pre and post multisensory stimulation. In a second controlled clinical trial (CCT) with *n* = 30 comatose head injury patients (at least 2 weeks from the trauma), the treatment consisted of 45 min (twice a day) visual, auditory, olfactory, cutaneous, kinesthetic and oral stimulation (six modalities) for a 1–3 months period (Kater, 1989). Outcome was defined as level of cognitive functioning (LCF) measured 2 weeks and 3 months after the trauma. In the third study (CCT), 12 traumatic brain-injured comatose patients (4–12 days after trauma) in the intervention group received 60 min (once or twice a day for up to 4 weeks) multisensory stimulation (visual, auditory, olfactory, tactile, gustatory, kinesthetic, and vestibular) (Mitchell et al., 1990). Outcome measures were GCS and total duration of coma. None of the three studies found any evidence of a therapeutic effect of multisensory stimulation programs in comatose brain injury patients (Lombardi et al., 2002). Despite these negative findings, some limitations of these studies need to be addressed: The duration of coma was quite short in all three studies while early rehabilitation patients frequently suffer from longer lasting DOC, such as UWS or MCS. Further, intervention (intensity and quality of multisensory stimulation) differed substantially between the three studies.

A more recent review focusing on MCS patients after traumatic brain injury included other stimulation techniques such as transcranial magnetic and deep brain stimulation (Lancioni et al., 2010). There is broad evidence that repetitive transcranial magnetic stimulation (rTMS) as well as deep brain stimulation (DBS) may be used for therapeutic purposes and that both types of stimulation interfere with cortical functions (Däuper et al., 2002; Rollnik et al., 2003). One comatose patient was treated with rTMS of the right dorsolateral prefrontal cortex (DLPFC) daily over 6 weeks (thirty sessions with 300 trains) demonstrating slight improvements of awareness (Louise-Bender Pape et al., 2009). The DLPFC is also the focus of rTMS in patients suffering from major depression to improve mood, fatigue and activity (Chen et al., 2013). Given that the thalamus plays a major role in consciousness and has been referred to as the gateway of sensory input, a bilateral DBS of the central thalamus has been tried in a few comatose patients, with only moderate effects (Yamamoto et al., 2005; Schiff et al., 2007). The review also identified more recent case reports focusing on multisensory stimulation in MCS or UWS describing the case of a 24-year old women close to MCS (Canedo et al., 2002). She had a brain injury 3 months before auditory, visual, and tactile stimulation was performed. By the eighth week she started to respond to tactile and auditory stimuli, by the tenth week, she started to communicate (Canedo et al., 2002). In another case, a 20-year old women with UWS was treated with a multisensory stimulation program (visual, auditory, tactile, gustatory and olfactory stimulation) 50 days after brain damage for 63 days (2-h sessions per day) (Bekinschtein et al., 2005). Soon after beginning of the program, the woman made some progress, e.g., following of simple commands (Bekinschtein et al., 2005). These case reports are only anecdotal and cannot replace controlled studies.

Multisensory stimulation is also the basis of the so-called “basal stimulation” which has been established in many German intensive care and early rehabilitation facilities (Menke, 2006). It comprises multisensory stimulation during the nursing process, e.g., somatosensory (initially touching hands, arms, shoulders or chest, body washing), vestibular (moving the head), oral (smell and taste of favorite food), vibratory (vibration of the chest or using an electric shaver), auditory (listening to familiar sounds and music), tactile (putting well known things like a tooth brush or a cup into the patient's hand) and visual stimuli (presenting pictures of relatives). There are, however, no controlled studies available. Basal stimulation is derived from the concept of enriched environment (LeWinn and Dimancescu, 1978). Interventions are not as standardized as in the studies mentioned above with respect to intensity or quality of stimulation (Kater, 1989; Mitchell et al., 1990; Johnson et al., 1993) and are a part of the nursing process.

Several pharmacological interventions have also been studied. The most promising results could be observed with the dopamine releaser amantadine in traumatic brain injury (TBI) patients (Wheaton et al., 2009). It is well known from Parkinson therapy that levodopa improves alertness (Bliwise et al., 2012). In a meta-analysis, 11 pharmacological treatments were investigated by 22 clinical studies, comprising 6472 TBI patients in the treatment groups and 6460 TBI controls. Only one dopamine releaser (amantadine) and 1 bradykinin antagonist (CP-0127 [Bradycor]) produced marked treatment benefits for a single measure of arousal (Glasgow Coma Scale) (Wheaton et al., 2009).

## Music therapy in neurological early rehabilitation patients with DOC

According to the G-DRG (German Diagnosis Related Groups) system, music therapy may be a part of the therapeutic concept in neurological early rehabilitation^1^. Music therapy in neurological rehabilitation has a long tradition in Germany (Muthesius, 2003). Although controlled studies are lacking, about 29% of neurological rehabilitation facilities in Germany have reported to offer music therapy (Jochims et al., 2003). However, most of these therapies refer to the use of live music and singing, for instance, involving the patient as an active part. This form of music therapy makes more sense in aware, conscious patients, not in neurological early rehabilitation subjects suffering from DOC.

However, it has been suggested that music therapy could be used to “communicate” with individuals suffering from DOC and motor disabilities (Magee, 2007). As the auditory modality has been found to be particularly sensitive in identifying responses indicating awareness, a standardized protocol for intervention or measuring patient responses within the music therapy setting has been developed, the so-called “music therapy assessment tool for low awareness states” (MATLAS) (Magee, 2007) and its advanced version “music therapy assessment tool for awareness in disorders of consciousness” (MATADOC) (Magee et al., 2014). MATLAS and MATADOC may be used for MCS or UWS patients and comprise items which rate behavioral responses to sensory stimulation (Magee, 2007; Magee et al., 2014). The 14 items of the MATADOC are: “responses to visual stimuli, responses to auditory stimuli, awareness of musical stimuli, response to verbal commands, arousal, behavioral response to music, musical response, vocalization, non-verbal communication, choice-making, motor skills, attention to task, intentional behavior, emotional response” (Magee et al., 2014). As an example, the item awareness of musical stimuli is rated from 0 (“no observed response”) to 5 (“showed consistent interactive responses within musical exchange”) (Magee et al., 2014). The MATADOC has been validated in a small study enrolling only *n* = 21 DOC patients after traumatic, hypoxic-ischemic, hemorrhagic brain damage or viral infection (Magee et al., 2014). In a prospective, non-controlled study with repeated measurements, internal consistency, inter-rater and test-retest reliability and dimensionality were examined (Magee et al., 2014). The five-item scale showed an internal reliability of α = 0.76 (Magee et al., 2014). Corrected item-total correlations were all above 0.45, inter-rater intra-class correlations (ICCs) ranged from 0.65 to 1.00 and intra-rater ICCs from 0.77 to 0.90 (Magee et al., 2014). The study showed that diagnostic outcomes had 100% agreement with a validated external reference standard (Magee et al., 2014). However, validity and reliability of the MATADOC should be examined enrolling a larger and homogenous cohort of patients.

Active music therapy (“playing”) has been tried in severely brain-injured patients who were already able to cooperate to a certain extent (Formisano et al., 2001). Therapy consisted of musical improvisation between patient and therapist by singing or by playing different musical instruments, according to the vital functions, the neurological condition and motor abilities of the patients (Formisano et al., 2001). *n* = 34 brain-injured patients with a mean coma duration of 52 days and a mean interval from coma onset to the beginning of rehabilitation of 154 days had been enrolled (Formisano et al., 2001). Results showed a significant improvement of the collaboration of the severely brain-injured patients and a reduction of undesired behaviors such as inertia or psychomotor agitation (Formisano et al., 2001).

In one case report, music therapy has been tried in a cerebral hypoxia patient whose diagnosis of UWS was contradicted by purposeful responses within the music therapy assessment, changing the diagnosis to MCS (Magee, 2005). This case illustrates the potential role of music therapy in assisting with diagnosis of patients with DOC (Magee, 2007; Magee et al., 2014). Thus, music therapy might provide a medium which does not rely on language, is non-evasive and elicits emotional responses in these patients (Magee, 2005).

A recently published study applied preferred music exposure in a larger cohort of patients with either UWS or MCS compared to healthy controls (O'Kelly et al., 2013). The neurophysiological and behavioral study was undertaken comparing electroencephalogram (EEG), heart rate variability, respiration and behavioral responses of 20 healthy subjects with 21 individuals with UWS or MCS (O'Kelly et al., 2013). Healthy subjects and patients were presented with live preferred music and improvised music entrained to respiration (procedures typically used in music therapy), recordings of disliked music, white noise, and silence (O'Kelly et al., 2013). ANOVA tests indicated a range of significant responses across healthy subjects corresponding to arousal and attention in response to preferred music including concurrent increases in respiration rate with globally enhanced EEG power spectra responses across frequency bandwidths (O'Kelly et al., 2013). Whilst physiological responses were heterogeneous across patient cohorts, significant *post hoc* EEG amplitude increases for stimuli associated with preferred music were found for frontal midline theta in six UWS and four MCS patients and frontal alpha in three UWS and four MCS subjects (O'Kelly et al., 2013). Furthermore, behavioral data showed a significantly increased blink rate for preferred music within the UWS cohort (O'Kelly et al., 2013). Two UWS patients showed concurrent changes across measures indicative of discriminatory responses to both music therapy procedures (O'Kelly et al., 2013). The results also suggested that music may be used to distinguish MCS from UWS (O'Kelly et al., 2013). However, due to the heterogeneity of the patient group, the study may rather be considered as a case study than a systematic investigation (O'Kelly et al., 2013).

## Discussion

Music therapy, in particular music listening, may be used in patients with DOC as part of an enriched environment setting during neurological early rehabilitation (Jochims et al., 2003; Muthesius, 2003; Menke, 2006; Lippert-Grüner et al., 2007; Magee, 2007; O'Kelly et al., 2013). It has been shown that music listening induces a broad activation of several complex neuronal networks and elicits emotional processes in the brain (limbic system) in alert subjects (Peretz and Zatorre, 2005; Altenmüller and Schlaug, 2013). It is evident from neuroimaging studies that even patients suffering from UWS (previously known as vegetative state) show emotional processing of auditory or visual information (Coleman et al., 2007; Eickhoff et al., 2008; Yu et al., 2013). Thus, it seems reasonable to believe that music listening, in particular listening to familiar music [as performed in the “basal stimulation” concept (Menke, 2006)], may be a powerful stimulator in the therapy of patients suffering from DOC. However, there is a considerable lack of evidence. Some controlled studies on multisensory stimulation are available, but they could not prove its efficacy in UWS or coma (Lombardi et al., 2002). In addition, there is only limited evidence that music therapy may be applied when subjects regain consciousness as a means of non-verbal communication or as a diagnostic tool to distinguish between UWS and MCS (Magee, 2005, 2007; Magee et al., 2014). Compared to patients with DOC, there is by far more evidence for the efficacy of “active” music therapy in alert neurological rehabilitation patients, in particular in motor rehabilitation (Altenmüller and Schlaug, 2013).

The therapeutic potential of music therapy in patients with coma, UWS or MCS in neurological early rehabilitation merits further investigation. Currently, there is (like with many other interventions in neurological rehabilitation) a considerable lack of evidence. Proof-of-principle and open-label studies, followed by controlled trials on this topic are strongly encouraged to improve the evidence-base of music as a therapeutic tool in neurological early rehabilitation patients with DOC.

Future studies should consider the following:

1. Precise clinical definition and homogeneity of the patient cohort with respect to the quality (coma vs. UWS vs. MCS), duration (rather weeks to months than days) and cause (traumatic vs. non-traumatic) of DOC.
2. Standardized intervention protocol: quality (e.g., familiar/preferred music, live or recorded) and intensity of music listening (frequency, duration of intervention). Studies should also consider a multisensory stimulation (music with or without other modalities).
3. Use of valid outcome parameters, in particular clinical (e.g., GCS, low- and high-level signs of consciousness) over a longer observation period (weeks to months).
4. Use of neurophysiological (EEG) as well as vegetative parameters (heart rate, respiration, skin conductance) to monitor physiological responses to music.
5. If available, use of neuroimaging to confirm diagnosis and to demonstrate responses and functional changes in the patients' brains.

### Conflict of interest statement

The authors declare that the research was conducted in the absence of any commercial or financial relationships that could be construed as a potential conflict of interest.

## Footnotes

^1^http://www.g-drg.de/cms/G-DRG-System_2014
